# Barriers to accessing and receiving mental health care for paid and unpaid carers of older adults

**DOI:** 10.1111/hex.14029

**Published:** 2024-03-25

**Authors:** Clarissa Giebel, Laura Prato, Sue Metcalfe, Hazel Barrow

**Affiliations:** ^1^ Department of Primary Care and Mental Health University of Liverpool Liverpool UK; ^2^ NIHR Applied Research Collaboration North West Coast Liverpool UK

**Keywords:** carers, dementia, inequalities, mental health, social care

## Abstract

**Aim:**

The aim of this qualitative study was to explore the barriers and facilitators to accessing and receiving mental health care for paid and unpaid carers of older adults.

**Methods:**

Unpaid and paid carers for older adults in England were interviewed remotely between May and December 2022. Participants were asked about their experiences of mental health needs and support. Reflexive thematic analysis was used to analyse the data.

**Results:**

Thirty‐seven carers participated (*n*_paid_ = 9; *n*_unpaid_ = 28), with the majority caring for a parent with dementia. Thematic analysis generated four themes: lack of healthcare support, social care system failing to enable time off, personal barriers and unsupportive work culture. Healthcare professionals failed to provide any link to mental health services, including when a dementia diagnosis was received. Structural and organisational barriers were evidenced by carers being unable to take time off from their unpaid caring duties or paid caring role, due to an absence of social care support for their relative.

**Conclusions:**

This is the first study to have explored the barriers to mental health care and support for paid and unpaid carers for older adults and suggests that structural, organisational and personal barriers cause severe difficulties in accessing required support to care for older relatives, services users and residents.

**Public Involvement:**

Two unpaid carers aided with the development of topic guides, data analysis, interpretation and dissemination. Both were supported and trained to code anonymised transcripts.

## 1 INTRODUCTION

Across the United Kingdom, 13.6 million unpaid carers^1^ provide substantial care for family members/friends, taking economic pressure off government‐ or local authority‐funded social care. For dementia alone, 700,000 people are caring in an unpaid capacity for a person with dementia across the United Kingdom. Dementia is an ongoing and growing public health concern, affecting around 900,000 people, and reaching one million by 2024.^2^

Before the coronavirus disease 2019 (COVID‐19) pandemic, many unpaid carers for older adults received little structured wellbeing or emotional support^3^, ^4^ for the impact of their caring role. Unpaid carers for dementia have high levels of unmet needs, ranging from limited opportunities to socialise, restricted knowledge of dementia and poor support with financial and legal issues leading to substandard physical and mental health.^5^, ^6^, ^7^ In a recent Australian survey of 169 carers for people with dementia, for example, Mansfield et al.^6^ reported that emotional wellbeing was one of the most common unmet needs. Often, they fail to focus on their own wellbeing and instead focus all their efforts on providing care for their relative with dementia.^8^ This is amplified by a lack of external support, such as paid home care or respite care, to provide time away from caring duties.^9^ Poor and unsupported mental health is not only detrimental to the unpaid carer but is also associated with negative outcomes for the person with dementia who is being cared for.^5^

The paid social care workforce is also struggling with severe unmet support needs and mental health issues.^3^ Paid carers for older adults, including those working in home care and care home settings, often struggle with burnout and increased workloads without any support or adequate training provided.^10^ One in five residential care workers are found to live in poverty in the United Kingdom,^11^ thus placing further personal pressure on care home staff. Whilst care home staff in the United Kingdom felt unsupported before the pandemic, COVID‐19 has exacerbated the levels of stress and work demands placed on care home staff.^12^, ^13^ This further highlights the need for adequate and easily available mental health support. However, no evidence to date has explored the barriers, or facilitators, to accessing mental health support for paid, or unpaid, carers of older adults.

Unpaid carers have dealt with significant changes, challenges and new pressures since the pandemic. With social care and social support services closing down and remaining closed or being delivered remotely since the beginning of the pandemic,^9^, ^14^ unpaid carers took on additional caring tasks which led to increased levels of carer burden and poorer mental health.^15^, ^16^ Similar to paid carers, there is little to no evidence on their long‐term mental health needs, and specifically on the experiences of accessing mental health care.

Whilst there is growing evidence on inequalities in accessing dementia care for unpaid carers,^4^, ^17^, ^18^ little attention has been paid to potential barriers, or facilitators, for both paid and unpaid carers of older adults in accessing support for their mental health. Considering the close relationship between accessing care services for a relative and mental health for unpaid carers,^9^ and similarity in the care provided by unpaid and paid carers, the aim of this qualitative study was to explore the barriers and facilitators to accessing and receiving mental health care for paid and unpaid carers of older adults.

## 2 METHODS

### 2.1 Participants and recruitment

Three carer groups were eligible to participate. Unpaid carers aged 18+ caring for an older adult (aged 65+) with or without dementia currently or who have cared for someone with dementia in the past 5 years, and resided in the United Kingdom, were eligible to take part. We included paid carers who were working in home care and residential care. Paid carers had to be aged 18+ and be working in the social care sector (care home or in‐home care) in the United Kingdom. Given the lack of evidence on barriers to mental health support and care for adult social care staff working with older adults, this exploratory study sought to include staff from different care settings, whilst recognising the differences in these settings and level of care demands.

Carers were recruited via convenience sampling, by approaching social care and support services and Third Sector organisations for older adults, dementia, carers, as well as care home and home care organisations using flyers. The flyer was also shared via the public Liverpool Dementia & Ageing Research Forum and via social media. Interested carers could contact the principal investigator via email, who forwarded the study information sheet and checked eligibility. Interested participants could either confirm their interest after having read the information sheet or were emailed again after 1 week to discuss their potential interest.

Ethical approval was obtained from the University of Liverpool ethics committee (Ref.: 11045) before the study began.

### 2.2 Data collection

Topic guides (one for unpaid carers and one for paid carers) were co‐produced with three public advisers (unpaid carers) (see Section 2.4) as part of the research team, and are included in Appendices A and B. Topic guides included questions on carers' experiences of mental health and wellbeing, any unmet needs, available and accessible support, barriers to accessing care and recommendations for making mental health care and support more equitably accessible.

Semistructured interviews were conducted remotely via Zoom between May and December 2022 by a research associate trained in qualitative data collection. Before the interview, verbal informed consent was audio recorded. Audio files were transcribed by two trained transcribers from the University of Liverpool, and anonymised.

### 2.3 Data analysis

Data were analysed using reflexive thematic analysis.^19^ Each transcript was coded by a research team member with two of those transcripts coded by one public adviser and unpaid carer. The public adviser received support and training from the researchers, who are experienced in conducting qualitative research in dementia and ageing. The six steps of thematic analysis were employed, by firstly familiarising oneself with the data by reading the transcripts, to then generating codes individually. The team met twice to discuss the codes and jointly search for, review and define themes, to then write this up and discuss the generated themes with the second public adviser who was not involved in the coding of data.

### 2.4 Public involvement

Three public advisers (two unpaid carers and one home care and day care centre staff) were involved in the development of the topic guides and provided feedback on study documents. One unpaid carer was trained and supported to code two anonymised transcripts, and helped shape the formation of codes and themes. The public adviser working in a home care and day care agency left the study throughout without further email communication, and thus both unpaid carers were involved in interpreting the findings and contextualising the findings within their lived caring experiences. Both unpaid carers read drafts of the manuscript and provided comments, and also wrote a lay summary of the findings which will be shared via the NIHR Applied Research Collaboration North West Coast and with all study participants. Public advisers were reimbursed for their time according to NIHR guidance.

## 3 RESULTS

Thirty‐seven carers for older adults participated in the study (*n*_paid_ = 9; *n*_unpaid_ = 28). Carers were mostly female (*n* = 32; 86.5%). Unpaid carers were on average 57.4 (±10.1) years old (range: 28–78), caring predominantly for a parent (*n* = 22; 78.6%). Fewer carers cared for their spouse (*n* = 5; 17.9%) or for their sibling (*n* = 1; 3.6%). Paid carers were on average 48.3 (±10.2) years old (range: 31–63). The majority of all carers were from a White British background, with three carers from a non‐White ethnic background (*n* = 3).

### 3.1 Qualitative findings

Using reflexive thematic analysis, four overarching themes were generated: lack of healthcare support, social care system failing to enable time off, personal barriers and unsupportive work culture. Quotes are included in Table 1 by theme and subtheme.

**Table 1:** Quotes by themes and subthemes.

Themes and subthemes	Quote
*Lack of healthcare support* Lack of guidance	‘I don't think so I think it's just knowing where would the best place to go, I think I mentioned the GP which would be probably my first port of call if I felt like I was having a problem. But yes the care services that have been involved so far have dealt with my dad, they haven't dealt with the family as a whole’. (Participant 7) ‘At the beginning there was no support really for either of us, we were told that Husband would, he when he had his diagnosis we were told he would just be discharged back to his GP and that was it’. (Participant 9) ‘But I never felt awfully supported by our GPs, I never felt, I mean I did speak to them, I did go to them, but I never felt that we got the help or support that we needed. The carer's centre was great and I used to be able to go to them’. (Participant 14) ‘I have to say that there is a little in place to do that. I can always call him offload, I can call HR and they will chat with me. But I have raised it in the past, there is nothing that we do for staff wellbeing. We are not offering any contacts to you know independent counselling or somebody to talk to, I have raised that in the past so I would say that's enough in my organisation for me or the staff’. (Participant 36) ‘Alzheimer's Society have supported us in that, Admiral Nurses have supported us in that and they've told us to do things that we hadn't thought of and they're, my God I didn't know that you know and that's really helped in you know making us more positive as well and having support workers around that you know you can call up’. (Participant 13) ‘Emotionally there is nothing there, occasionally I have tried ringing a couple of the carer charities and they have no answers, they have, there's nothing there. At the risk of sounding really horrible I have no idea what the hell the carer charities do’. (Participant 12)
Time‐pressured healthcare appointments	‘I've spoken to my doctor about it, they'll talk to you but as they say there's not really a lot we can do for you so you know, if you need to talk ring us, but you get a 5‐min appointment and that's it, 5 min and you're off again aren't you’. (Participant 05)
Reaching crisis point	‘But it often had to get to crisis point before anything was done, by which point I would be yeah at the end of my ability to cope’. (Participant 09) ‘I did have good support from the Admiral nurse she was really good. But again, I think had I had it earlier in the, you know, the journey I might not have felt like I couldn't cope which is what happened in the end’. (Participant 09) ‘So you try to ring them up and after you've spent 45 min listening to you know your call is important blah blah blah you eventually get through. They put you through to somebody else and all you get is a voicemail and you get no response’. (Participant 17)
*Social care system failing to enable time off*	‘I feel that I definitely did not get the support to allow me to regularly have days away. I had to really like fight for days off and the other problem is that with respite even when we did get respite my mum was very very reluctant to go to respite’. (Participant 03) ‘In terms of the social care, part of the same kind of thing when they were providing carers to come and help with my dad at one stage for four times a day, but [on] four occasions they would send these carers in. They were unreliable, they wouldn't turn up at the correct time or even within, they were supposed to come’. (Participant 17) ‘I had a carer's centre I used to go to, but when I was out at the carers centre someone, my neighbour would phone me and say your mums got out and I would have to leave so that became impossible’. (Participant 14) ‘I think all of those and particularly shift patterns and financial, because everybody, I mean when you're working in this sort of job you pretty much live week to week, you can't afford to just take time off work to go to things like that’. (Participant 33)
*Personal barriers* Failure to acknowledge need for support	‘But I wasn't aware of it I just thought I was just having a really stressful day and had been a really stressful time. So I did suffer but I didn't I didn't know so I didn't ask for help because I didn't know I needed it’. (Participant 18) ‘Asking for help, realising that you absolutely must have the help and yeah and acknowledging that situation, it's difficult’. (Participant 19)
Lack of informal support network	‘When I was literally breaking down, I couldn't ask people for help because I actually couldn't speak you know I couldn't, it wasn't even so much as couldn't I rarely cry because I've always lived alone so there's no point crying because there's no one to you know see to you. So you kind of condition yourself not to cry as a reaction to stress or distress. But believe me I have cried on several times not in sight of my dad of course but I have cried. But after about 4 months I was at a point where I actually couldn't even really speak to ask people for help or tell them I needed help because I was just so overwhelmed.” Participant 17“I think there are some great support mechanisms out there, you've got TIDE which are a really good organisation, you'll have heard of them because that's how I got on to this but for me I just couldn't access it because Husband wouldn't allow me to do it. You know I couldn't sit, these have these weekly, monthly zoom chats.’ (Participant 09)
Duty to care	‘I haven't been able to do that because I don't want to give a feeling that I am absconding from what I'm supposed to be doing for my mum’. (Participant 06) “Flashbacks they're most difficult, the difficulty with mum was that she had a very angry and difficult journey, she was scared and aggressiveness because she was afraid of what was going on in her mind with her illness and she refused all outside care and assistance so it was just me and my mum 24/7 in those last years’. (Participant 14)
*Unsupportive work culture*	‘I mean there's lots of posters, wellbeing hubs and download the latest app and all of that well that doesn't do it, that's just another load of stress and its impersonal, it might work for some people wouldn't work for me. I think it's dealing with things at the time rather than let it build up and I think there should be a more supportive network in each facility. But it's getting over this barrier of being able to say how you feel without recriminations’. (Participant 31) “I can't see that on senior level they seem to be either strapped for time or too wrapped up with what they need to be doing and disciplining people for absolutely ridiculous reasons. But I do think you get it from line management and colleague support. I can't see any external support, I have noticed a few noticeboards saying wellbeing but it's not promoted very well, I've just seen it in the canteen’. (Participant 34) ‘We do have a really supportive network so you know obviously I'm a line manager, [name] is the manager and she's, to be fair she is incredibly supportive to everybody. It doesn't matter who it is, she's accessible now all day sometimes to her detriment, you know, because she doesn't, you know she's always there’. (Participant 32) ‘So, we should advise well go to your GP and seek help if you can't we have this in place. I think the easiest we should do is post them to independent counselling, somebody to talk to. We do have occupational health which can help with physical problems if they can't cope with work and we can reassign them but the mental health wellbeing is not supported enough, looked after enough’. (Participant 36) “The care home I work with is very well managed. The management are very supportive you know there's ongoing training there so you know you are constantly updated and if you have any kind of issues you can always go and talk to someone about them’. (Participant 08) ‘I can't see that on senior level they seem to be either strapped for time or too wrapped up with what they need to be doing and disciplining people for absolutely ridiculous reasons. But I do think you get it from line management and colleague support. I can't see any external support, I have noticed a few noticeboards saying wellbeing but it's not promoted very well, I've just seen it in the canteen’. (Participant 34) ‘We do have a really supportive network so you know obviously I'm a line manager, [name] is the manager and she's, to be fair she is incredibly supportive to everybody. It doesn't matter who it is, she's accessible now all day sometimes to her detriment, you know, because she doesn't, you know she's always there’. (Participant 32)

Abbreviation: GP, general practitioners.

### 3.2 Lack of healthcare support

#### 3.2.1 Lack of guidance

Both paid and unpaid carers received little to no guidance on accessing mental health care. Many carers caring for a relative with dementia received no support for their own needs at the time of the dementia diagnosis, leaving carers with little knowledge or awareness of their right to access support for their mental health. This was particularly amplified for unpaid carers of older adults without dementia, but who were suffering from frailty and/or other comorbidities. For some unpaid carers, their relative became in need of care and thus needed to see a healthcare professional for various health issues.

Unlike unpaid carers, paid carers were not in contact with healthcare professionals regarding a relative's health issues, and thus had even less opportunity to engage with a healthcare professional about their needs. Instead, they provide care for vulnerable older adults often without any support. Many paid carers reported that leaflets to discuss one's mental health were the only visible recognition of mental health support, without easy access to external mental health care via the NHS.

Both paid and unpaid carers had to seek mental health support themselves without being offered or informed about these. The lack of clear guidance from healthcare professionals, and in the work environment, created barriers for many carers in accessing care.

Some participants identified that Third Sector organisations provided significantly more support than health and social care professionals. Both social care and healthcare professionals were frequently characterised as not listening to carers in contrast to more attentive and supportive Third Sector agents. However, there were opposing opinions expressed by some carers who were critical of the Third Sector.

#### 3.2.2 Time‐pressured healthcare appointments

As suggested by carers, general practitioners (GPs) could facilitate access to mental health support services through proactive practice. However, GPs could also function as barriers to accessing appropriate support where carers experienced rushed appointments, or where there was an absence of individualised support services available in the local area.

#### 3.2.3 Reaching crisis point

Unpaid carers identified that mental health support was sometimes offered during periods of crisis but not earlier in the carer journey. One unpaid carer described being offered support only after their relative was admitted to a care home and expressed regret that it had not been available sooner (Participant 09). Where mental health support was offered, unpaid carers sometimes experienced long waits in accessing care due to very limited availability and a multitude of technological barriers. One carer discussed waiting 8 months for support and others discussed the frustration and futility of ringing phone numbers for long periods with no response. However, admiral nurses were identified as having the potential to advert crisis points if accessed early enough in the carer journey.

### 3.3 Social care system failing to enable time off

Both paid and unpaid carers reported difficulties in accessing mental health care due to limited available free time. Unpaid carers often struggled to access sufficient, or at times any, social care and support services for their relative or for themselves, including day care, respite care or support groups. As a result, unpaid carers were taking on all or nearly all caring duties, leaving little to no time for any leisure activities or time off from caring. This acted as a notable barrier to accessing mental health support. Further to this, where respite care was available, carers often felt it was of substandard quality and were reluctant to access it. Carers who did receive adequate respite care valued the reliability of provision.

Even when external social support was available to allow carers a break, this could be affected by emerging caring needs for their relative. When attending support services, carers may be called back home by neighbours or other acquaintances due to their relative requiring care and supervision. This was amplified by most carers caring alone for their relative.

Similarly, paid carers reported difficulties in accessing mental health services due to the need to access these services outside of working hours. Services such as therapy were experienced to only be provided during the day time, when some paid carers were unable to attend. In addition, regardless of the timings of care services, paid carers were expected to access mental health support within their own time, thus unable to earn money during that period. This was identified as financially and logistically unviable.

### 3.4 Personal barriers

#### 3.4.1 Failure to acknowledge need for support

Unpaid carers experienced two significant personal barriers to accessing mental health support: a reluctance to acknowledge the need for support and a strong sense of personal duty to the person living with dementia. Unpaid carers reported finding it difficult to acknowledge the need for personal mental health support. Sometimes this was characterised as a reluctance to put one's own needs ahead of the needs of the person living with dementia. At other times, unpaid carers identified that depression played a role in the inability to recognise the need for mental health support. Fears around experiencing stigma and the potential impact of a mental health diagnosis on accessing insurance and future career progression were evident. Carers were often reluctant to acknowledge the need for support due to a desire to shield family members from the reality of the difficulties of caring. Instead, carers often attempted to uphold the image of a strong ‘front’.

#### 3.4.2 Lack of informal support network

Some carers had no support networks to discuss their experiences with. Many unpaid carers were the sole carer, whilst paid carers were also often struggling to receive peer support and discuss the emotional aspects of caring.

Whilst some unpaid carers were aware of non‐NHS mental health support systems, they were not always able to access these for various reasons. This included people cared for not enabling the carer any time off, and no external support to enjoy a break from caring to access support systems.

#### 3.4.3 Duty to care

Unpaid carers often felt a personal duty to care for their relative, and accepted distressing experiences and emotional burnout as part of their caring role. Some carers were the only relative living nearby who could or would provide care and thus felt more obliged to care for their parent. Love was identified as the motivating factor behind the duty to care, with carers identifying that they would do it all over again regardless of personal cost because of the strength of familial love.

### 3.5 Unsupportive work culture

Paid carers identified the challenges of accessing mental health support in work settings that were often unsupportive and lacking in both facilities and access to mental health services. They were unsure where or how to access mental health support. When services were available, gaining access was often characterised as a slow process. The support available was identified as being impersonal and frequently consisting of online resource access rather than face‐to‐face support.

Paid carers identified that management personnel were often too busy to facilitate mental health support or were unapproachable as individuals. Workplace culture often prevented individuals from seeking support for their mental health, due to bullying and a reluctance to acknowledge the impact of professional care work on mental health. A number of participants identified that there was a stigma attached to admitting mental health struggles in the workplace. Fears of future career reprisals if one spoke up about their mental health were discussed. However, some paid carers also reported positive experiences with work culture and managerial support, highlighting the advantage of good mental health and wellbeing.

In both care home and home care settings, the completion of tasks was prioritised above employee wellbeing. This was felt to be due to the absence of national recognition of the carer profession. The absence of professional infrastructure, such as a designated union or national governing body, meant the absence of an appropriate edifice to facilitate access to mental health support. Some paid carers experienced a positive working environment; however, where training and support were actively prioritised.

In some organisations, a change in workplace culture was required. Paid carers who did experience a supportive culture identified that managerial figures who embedded and normalised mental health support were integral to achieving this. Facilitating peer support that acknowledged carers as individuals, with lives outside of their work role, helped to ensure a workplace that acknowledged the need for mental health provision. Approachable managers who listened to staff concerns and were flexible with working patterns were vital. Furthermore, a managerial structure who were cognisant of the importance of time away from the workplace was crucial to enabling access to mental health support.

## 4 DISCUSSION

This appears to be one of the first studies to explore the barriers to accessing mental health support for paid and unpaid carers of older adults, highlighting both their shared and different experiences. The most common experience for both paid and unpaid carers of older adults in England seems to be the many barriers surrounding mental health and wellbeing support. These range from healthcare professional‐initiated barriers to the social care system itself, personal barriers and unsupportive work cultures. Whilst these barriers were noted for many, some carers did experience supportive social networks, work cultures or healthcare professional interventions, indicating how barriers can be turned into facilitators.

Carers experienced a lack of guidance regarding how to access mental health care. Unpaid carers were mostly not considered as individuals in need of support by healthcare professionals and were not advised about necessary support they were entitled to, for example, at the point of the dementia diagnosis. Similarly, paid carers would often only view leaflets about mental health as opposed to being connected to psychologists or other healthcare professionals within their daily role. The lack of recognition of the value of carers has been reflected in recent reports by Carers UK^20^ and Skills for Care.^21^ The latter has reported a decrease in filled positions in adult social care in England in 2021/2022, compared to the previous year. Guidance needs to be clearly accessible, as existing time‐limited mental healthcare interventions for unpaid carers of people living with dementia, appear to be beneficial in reducing depressive symptoms and caregiver burden.^22^ This can be achieved by healthcare professionals actively offering information and support to unpaid carers during healthcare appointments with their relative.

For paid carers, structural changes need to be implemented to enable easier, free of charge and nonstigmatised access to mental health support. Corroborating existing evidence relating to the often‐amplified difficulties of working in adult social care in England since the pandemic,^16^, ^23^ this study reported similar barriers to accessing mental health support within the home care and care home sector. In corroboration with the findings of this study, a small number of paid carers experienced positive support systems. For some, this was only the case after having moved to a different care provider. Managerial support for the embedding of both organisational and external access to mental health support seemed to be key, which was linked to a supportive work culture enabling the sharing of difficult emotional experiences. Schwartz rounds could be one method to enable internal or externally supported safe spaces to discuss and share the emotional experiences of the job—from work demands, financial pressures and grief. To date, Schwartz Rounds have only been implemented with healthcare professionals.^24^, ^25^ However, the success of the Schwartz Rounds method, and the apparent need for better integrated mental health support, suggests that this might be a suitable option, with the potential to improve the mental health and well‐being of the adult social care workforce, and potentially retaining staff within the sector.

Providing integrated mental health support would also overcome an additional evidenced barrier of the social care system itself not enabling sufficient time off to access mental health support. This was evidenced for both paid and unpaid carers, with unpaid carers struggling to access suitable care services for their older relatives or relatives with dementia. This corroborates previous findings in the United States regarding increased carer strain and lack of access to dementia services,^26^ as well as growing research into inequalities in accessing and utilising dementia care.^4^, ^27^, ^28^ By enabling access to support services, such as day care and paid home care, unpaid carers would not only likely experience reduced levels of mental health problems,^9^ but also find the necessary time to access mental health services and address their unmet needs. Considering that mental health needs are evident in many carers consultations,^6^, ^7^ the social care system can act as a facilitator to improve mental health, showcasing again the inter‐relationship between mental health (and thus health care) and social care.

Where all other structural and organisational barriers are addressed, personal barriers to seeking mental health support may remain though. For unpaid carers, this often involved caring out of duty for their parent or spouse, or being the only family member who provides care. This could place additional burden upon oneself, as opposed to seeking out support. Whilst caring out of duty could also be motivated by positive factors, overall, it left many carers to not seek out external support leading to increased levels of carer burden with increased caring demands. These evidenced personal barriers are reinforced by previous research into the unmet needs of unpaid carers. Schoelzel‐Dorenbros et al.^29^ highlighted self‐reported barriers of unpaid carers for people with dementia when trying to access support, including the limited assistance regarding the management of dementia symptoms, socialising and information, and the inability to prioritise their own mental health and well‐being. Admiral Nurses may provide one avenue of support to facilitate discussions about carer mental health, although clearer guidance for Admiral Nurses on clearly addressing carer mental health would be required.^30^ In contrast, paid carers in this study were able to identify their own need for mental health support and were proactive in seeking support but were hampered by the limited availability and absence of clear signposting (organisational and structural barriers). This suggests that for paid carers in particular, addressing structural and organisational barriers is key to promoting access to mental health support.

### 4.1 Limitations

Whilst this appears to be the first study to explore the barriers to accessing and utilising mental health care for paid and unpaid carers of older adults, and provides novel insights into existing barriers, there are some limitations to this study. First, paid carers from the home care and care home sector were challenging to recruit, resulting in a larger proportion of unpaid carers. This may have been the result of increased work demands in the social care workforce, especially since the COVID‐19 pandemic.^16^ Future research needs to specifically target a larger sample of home and care home staff. Second, the sample lacks ethnic diversity, with three carers being from a non‐White (specifically Black African or Asian British) background. Whilst we evidenced stigma in the care sector regarding mental health, the issue of stigma may have also featured more strongly with a more ethnically diverse carer sample, as mental health is often highly stigmatised in minority ethnic groups.^31^, ^32^, ^33^, ^34^ Thus, future research needs to specifically focus on carers from diverse ethnic backgrounds to capture more representative viewpoints, including understanding the cultural variations in and reasons for caring for older relatives.

## 5 CONCLUSIONS

This is the first study to have explored the barriers to mental health care and support for paid and unpaid carers for older adults, and suggests that structural, organisational and personal barriers cause severe difficulties in accessing required support to care for older relatives, services users and residents. Both the social care system and care providers need to actively embed mental health and peer support to facilitate free and easily accessible assistance to enable the discussion of emotional issues related to caring. By normalising distressing emotional experiences as a result of the caring role, and accepting discussion of these issues, staff could be supported better. For unpaid carers, this involves greater recognition of their caring role when attending healthcare professional visits with their relative. Whilst these recommendations for clinical and social care practice can start addressing the barriers to mental health care, further research is required to specifically understand barriers for carers from minority ethnic backgrounds.

## AUTHOR CONTRIBUTIONS

**Clarissa Giebel**: Conceptualisation; investigation; funding acquisition; writing—original draft; methodology; writing—review and editing; formal analysis; data curation; supervision. **Laura Prato**: Writing—review and editing; formal analysis. **Sue Metcalfe**: Conceptualisation. **Hazel Barrow**: Formal analysis; conceptualisation.

## CONFLICT OF INTEREST STATEMENT

The authors declare no conflict of interest.

## Data Availability

The data that support the findings of this study are available on request from the corresponding author. The data are not publicly available due to privacy or ethical restrictions.

## APPENDIX A

**Topic guide for unpaid carers**

1. Can you tell me about your caring experiences for your relative (with dementia)? How long have you been caring?
2. What is/has caring (been) like for you? If you were to describe your emotions surrounding the caring experiences for me please.
3. Have you experienced that caring for your relative has impacted on your social life? If so, how?
4. *Current carers*: Do you feel you get the support you need as an unpaid carer? If not, what type of support would you need?
  *Former carers*: Do you feel you received the support you needed, and still do today? If not, what type of support would you need or have needed in the past that you did not get?
5. At any point during your caring experiences, have you felt that your mental health and well‐being was suffering? If so, have you approached someone about this and received support?
  *Additional question*: Have you approached a health care professional about your mental health?
6. What are your experiences of dealing with health and social care professionals about your relative's diagnosis? Has this in any way impacted on how you may have dealt with your own mental health and well‐being?
7. What do you think could be done to make the caring experiences less stressful?
8. How has the pandemic affected your ability to care for your relative and your well‐being?
9. Was there anything positive in your caregiving journey (so far) that has supported your mental well‐being and was positive?
10. Do you feel valued as a carer and that your views and opinions are being listened to and acted on?
11. Is there anything else you would like to add that we haven't discussed yet?

## APPENDIX B

**Topic guide for paid carers**

1. Can you tell me about your working experiences in the care sector? How long have you been working in care homes/paid home care and what has it been like?
2. What type of training have you received for your job? If you have moved between organisations/care homes, have you received updated training?
  *PROMPT: Knowledge about accessing training*
3. Reflecting on the wider working conditions in the care sector, have you had more positive or negative experiences? Can you share with us some examples of your experiences please?
4. Has there been any situations in work recently that you have felt you need support with and if so, what have they been? If you need support, do you have a supportive network or a go‐to person within your organisation? What would you do if you got distressed? How do you think the organisation would support you with your distress?
5. Do you feel that you receive enough support on the job and with any other issues emerging from caring for vulnerable people? (e.g. operational issues like PPI, moral injury, mental health issues, bereavement or grief issues or CPD requirements).
6. What has it been like working in the care sector during the COVID‐19 pandemic? How have you coped?
7. Since working in the care sector, how has your mental health and well‐being been? Do you feel that working in the care sector has affected/is affecting your well‐being? If so, how?
8. Have you ever sought out support for any mental health issues with a health care professional? Where and how supportive/successful was it?
  *PROMPT: How readily available was it?*
9. What do you think should be done to provide easily‐accessible support for mental health for social care staff? Can you think of any barriers to accessing this support (e.g. paid time to attend support, shift patterns, culture/stigma?)
